# Epidemiological trajectory of Mpox in Brazil, 2022–2025: evidence of geographic consolidation and syndemic burden

**DOI:** 10.1098/rsos.261003

**Published:** 2026-10-28

**Authors:** Gabriel do Nascimento Soares, Camila Innocente-Alves, Ana Luiza Marques, Tatiane da Silva Dal Pizzol, Fabrício S. Campos, Lucélia Santi, Walter Orlando Beys-da-Silva

**Affiliations:** ^1^ Faculdade de Farmácia, Universidade Federal do Rio Grande do Sul, Porto Alegre, Brazil; ^2^ Programa de pós-graduação em epidemiologia, Universidade Federal do Rio Grande do Sul, Porto Alegre, Brazil; ^3^ Instituto de Ciências Básicas da Saúde, Universidade Federal do Rio Grande do Sul, Porto Alegre, Brazil; ^4^ Centro de Biotecnologia, Universidade Federal do Rio Grande do Sul, Porto Alegre, Brazil; ^5^ College of Veterinary Medicine, Department of Public & Ecosystem Health, Cornell University, Ithaca, NY, USA

**Keywords:** mpox, epidemiology, Brazil, OpenDataSUS

## Abstract

Mpox has transitioned from outbreak to sustained transmission in Brazil, a country with high ecological and social connectivity. We analysed the epidemiological trajectory of mpox during 2022–2025 using 14 475 laboratory-confirmed cases reported by the Brazilian Ministry of Health. Our findings indicate geographical consolidation of transmission, with the highest incidence observed in the southeast and midwest regions. The epidemic disproportionately affects men (93.0%), particularly young adults, with transmission primarily associated with networks of men who have sex with men (69.1%). A substantial burden of co-infections was identified: 47.1% of reported cases occurred in persons living with HIV and 30.8% had syphilis. Clinically, most patients presented with cutaneous manifestations (91.3%). These findings highlight the convergence of epidemiological and social determinants shaping mpox transmission in Brazil. Strengthening integrated surveillance systems and ensuring equitable, stigma-free access to prevention and care are critical to reducing transmission and mitigating the risk of broader dissemination.

## 1. Introduction

Mpox is a zoonotic disease caused by monkeypox virus (also known as mpox virus; *MPXV),* a member of genera *Orthopoxvirus*, family Poxviridae [1,2]. Firstly identified in 1958 during outbreaks in captive primates, MPXV is endemic in tropical regions of Central and West Africa, where it has historically been sustained by cycles of zoonotic transmission [3]. Two major clades have been described: clade I (Central Africa), associated with more severe disease, and clade II (West Africa), typically linked to milder clinical outcomes. With the subdivisions of the clades, clade IIa is associated with zoonotic transmission, while clade IIb was first described during the 2022 outbreak, but was first identified in Nigeria in 2014, with outbreaks also identified in 2017 [4].

Transmission occurs through close contact with infected skin or mucosal lesions, respiratory secretions or contaminated materials [5]. During the 2022 global outbreak, sustained human-to-human transmission was documented, with a substantial proportion of cases associated with sexual contact networks [6]. Clinically, mpox is characterized by fever, lymphadenopathy and a vesiculopustular rash following an incubation period of approximately 5–21 days [7].

MPXV is a double-stranded DNA virus of approximately 198 kb, encoding ≈175 orthologous genes conserved across *Orthopoxviruses* [8,9]. The 2022–2025 global outbreak has been primarily associated with clade IIb, a lineage that emerged from Nigeria and was linked to widespread international dissemination [4,10]. As of early 2025, more than 180 000 confirmed cases have been reported across > 140 countries, with the Americas representing a major epicentre of transmission, particularly in Brazil and the USA [11].

Brazil reported one of the highest case burdens globally during the outbreak, reflecting its high population density, urban connectivity and social heterogeneity [12]. National surveillance efforts were rapidly implemented, including case notification systems, epidemiologic monitoring and integration with sexually transmitted infection (STI) programmes [13]. However, despite these efforts, sustained transmission has persisted beyond the initial epidemic wave, suggesting a transition from outbreak dynamics to more stable patterns of circulation [14].

Understanding the epidemiological trajectory of mpox in Brazil is essential for informing public health strategies and anticipating future transmission scenarios [15]. In this study, we analyse the temporal, demographic and geographic patterns of mpox in Brazil from 2022 to 2025, using nationwide surveillance data from OpenDataSUS to characterize the progression from emergence to epidemiological consolidation.

## 2. Methodology

The study was conducted using publicly available data from OpenDataSUS, the national open-access health database maintained by the Brazilian Ministry of Health (https://dadosabertos.saude.gov.br/dataset/mpox). Datasus, which is part of the Ministry of Health's SEIDIGI, is the entity responsible for technology and data integration within the SUS, operating with complete security and transparency. Its primary function is to provide reliable information to support health diagnoses, strategic decisions and the formulation of new public policies.

All records are anonymized prior to public release. In accordance with Brazilian regulations (resolution no. 510/2016) [16], studies based exclusively on publicly available, de-identified data do not require institutional ethics committee approval. Data were accessed on 20 January 2026.

We analysed laboratory-confirmed mpox cases reported between 2022 and 2025. To ensure diagnostic specificity, only cases classified as laboratory-confirmed according to the dataset definition (classi_fin = 1) were included. Suspected, discarded and inconclusive cases were excluded to minimize potential misclassification with other dermatologic conditions.

Demographic variables included age, sex, self-reported race/ethnicity, state of notification and sexual orientation. Clinical variables included reported signs and symptoms, clinical outcomes and documented co-infections (HIV, syphilis, gonorrhoea and chlamydia).

Data cleaning procedures were applied to ensure internal consistency. Records with implausible temporal intervals between symptom onset and notification (>60 days) were excluded. Additional filtering steps were performed to identify and remove or correct geographic inconsistencies.

Population data used to calculate incidence rates were obtained from the Brazilian Institute of Geography and Statistics (IBGE), based on the most recent national census estimates [17]. Descriptive analyses were performed to characterize temporal, demographic and geographic patterns. Continuous variables were summarized using medians and interquartile ranges, and categorical variables were expressed as proportions [18].

## 3. Results

### 3.1. Geographical distribution and population-adjusted analysis

The total number of mpox notifications from 2022 to 2025 was 71 355 cases; however, to ensure absolute diagnostic accuracy, strict inclusion criteria were applied. Initially, only laboratory-confirmed mpox cases (classi_fin = 1) were retained, which excluded 56 855 suspected, inconclusive or ruled-out records. The resulting subgroup of 14 500 confirmed cases then underwent a rigorous data cleaning phase to identify and address biological or chronological inconsistencies. At this stage, a 60-day limit was set for the interval between symptom onset and the date of notification; records exceeding this timeframe, as well as those with geographical inconsistencies, were excluded to preserve the integrity of the epidemic curve (*n* = 25 exclusions). This rigorous filtering process resulted in a final, robust cohort of 14 475 confirmed cases included in the analysis. All cases were reported in Brazil (table 1), and their evolution over the years is reported in figure S1 in the supplementary material.

**Table 1.** Sociodemographic and clinical characteristics of laboratory-confirmed mpox cases, Brazil, 2022–2025 (*n* = 14 475). Percentages were calculated excluding missing or unknown values.

variables	*n*	valid%
**age (years)**		
<18	440	3.0
18 to 29	4847	33.5
30 to 39	5990	41.4
40 to 49	2486	17.2
≥50	712	4.9
unknown/missing	0	–
**biological sex**		
male	13 437	93.0
female	1009	7.0
unknown/missing	29	–
**race/ethnicity**		
white	6096	48.3
mixed (Parda)	4693	37.2
black	1456	11.5
Asian	351	2.8
indigenous	24	0.2
unknown/missing	1855	–
**sexual orientation**		
homosexual	5168	69.5
heterosexual	1457	19.6
bisexual	815	11.0
unknown/missing	7035	–
**HIV status**		
negative	5841	52.9
positive	5204	47.1
unknown/missing	3430	–
**concurrent STIs**		
syphilis (positive)	1284	30.8
gonorrhoea (positive)	130	3.8
genital herpes (positive)	69	2.1
chlamydia (positive)	61	1.8

*valid% excludes unknown/missing data (complete case analysis).

The geographical distribution of mpox in Brazil (2022–2025) showed a marked concentration of cases in the southeast and midwest regions. In absolute numbers, São Paulo state accounted for the highest number of confirmed cases (*n* = 5843), followed by Rio de Janeiro (*n* = 1968) and Minas Gerais (*n* = 848). Additional states with substantial case counts included Goiás (*n* = 709), Paraná (*n* = 620) and Santa Catarina (*n* = 597) (figure 1).

**Figure 1.**
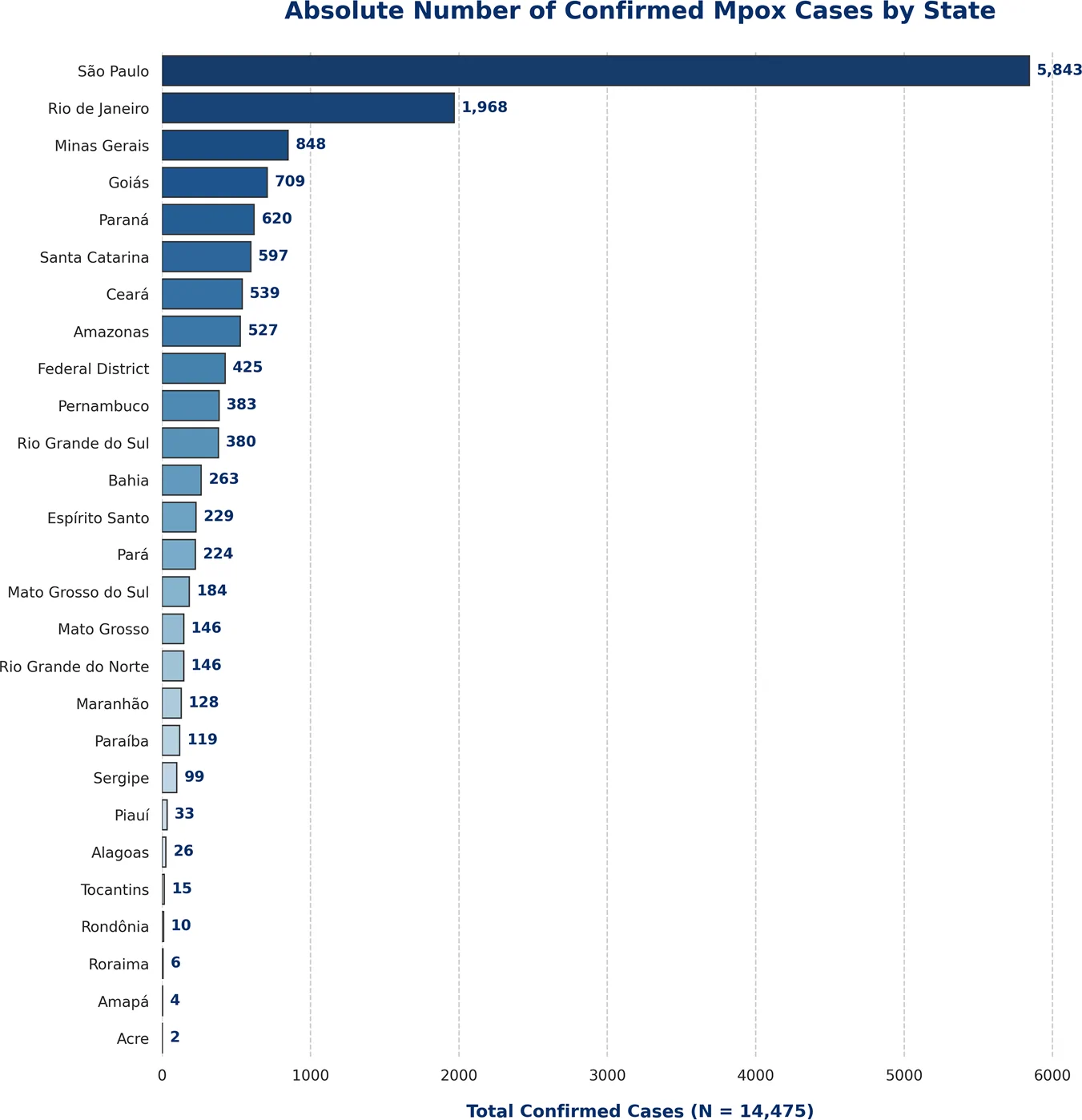
Distribution of laboratory-confirmed mpox cases across Brazilian states between 2022 and 2025 (*n* = 14 475). Data were obtained from OpenDataSUS (https://dadosabertos.saude.gov.br/dataset/mpox).

When population size was considered using national census data, incidence rates per 100 000 inhabitants revealed a more heterogeneous risk distribution across regions (supplementary material, table S1), with some states showing disproportionately higher incidence despite lower absolute case counts.

During the 2022–2025 period, the epidemic disproportionately affected males, who accounted for 93.0% (*n* = 13 437) of cases with available data. Cases were concentrated among young adults, particularly those aged 30 to 39 (41.4%) and 18 to 29 (33.5%). Among individuals with reported race/ethnicity, most self-identified as white (48.3%, *n* = 6096) or mixed-race/Parda (37.2%, *n* = 4693).

While the overall sexual orientation profile is presented in table 1, stratifying these data by biological sex reveals distinct epidemiological networks. Among male patients with a documented sexual orientation, the transmission was heavily concentrated within the MSM (men who have sex with men) community, with 72.0% (*n* = 4769) self-identifying as homosexual and 11.3% (*n* = 748) as bisexual. In stark contrast, among the female cohort with available data, the infection was predominantly associated with heterosexual contact, which accounted for 91.8% (*n* = 405) of the documented cases. This gender-based divergence highlights distinct vulnerability profiles and transmission pathways during the Brazilian outbreak.

A substantial burden of co-infections was observed. Among cases with available HIV status, 47.1% (*n* = 5204) were living with HIV. Screening for other STIs identified a high prevalence of syphilis (30.8%, *n* = 1284), whereas gonorrhoea (3.8%), genital herpes (2.1%) and chlamydia (1.8%) were less frequently reported.

### 3.2. Age and sex distribution

The age–sex distribution of confirmed mpox cases in Brazil demonstrated a pronounced asymmetry, with a marked predominance of cases among males across all age groups (figure 2). The highest concentration of cases occurred among young adults, particularly in the 25–29 and 30–34 age groups, forming a clear unimodal distribution centred in early adulthood.

**Figure 2.**
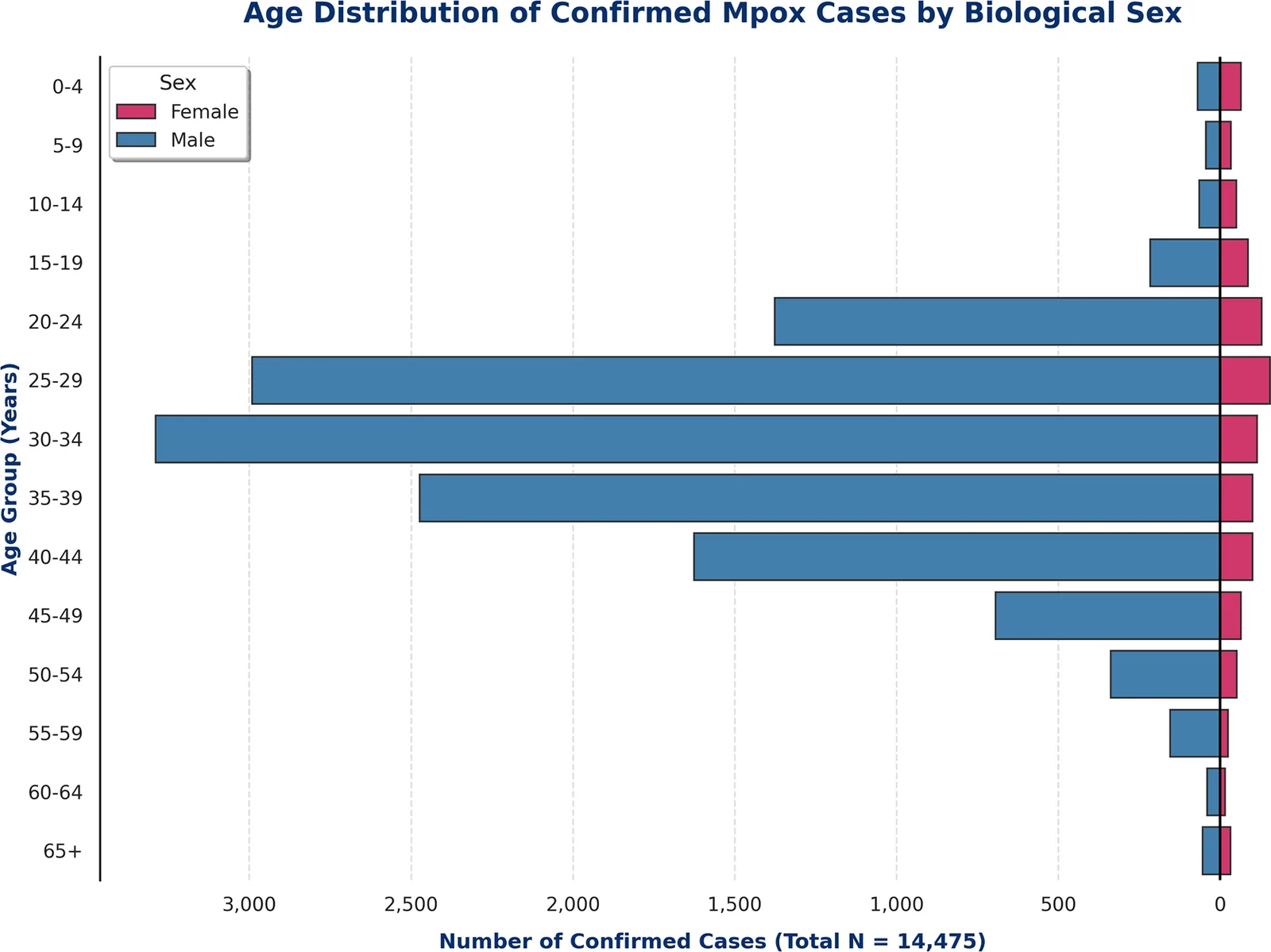
Age–sex distribution of laboratory-confirmed mpox cases in Brazil, 2022–2025 (*n* = 14 475). Data were obtained from OpenDataSUS.

By contrast, female cases were consistently low across all age strata, with no comparable peak observed. The distribution pattern highlights a strong age- and sex-specific clustering of cases, consistent with transmission concentrated within defined social and behavioural networks.

### 3.3. Clinical presentation

Cutaneous involvement was the predominant clinical feature, with skin rash reported in 91.3% of cases (*n* = 13 217) (figure 3). Fever was the most common systemic manifestation (58.3%; *n* = 8435), followed by lymphadenopathy (46.8%; *n* = 6774) and genital lesions (45.0%; *n* = 6510).

**Figure 3.**
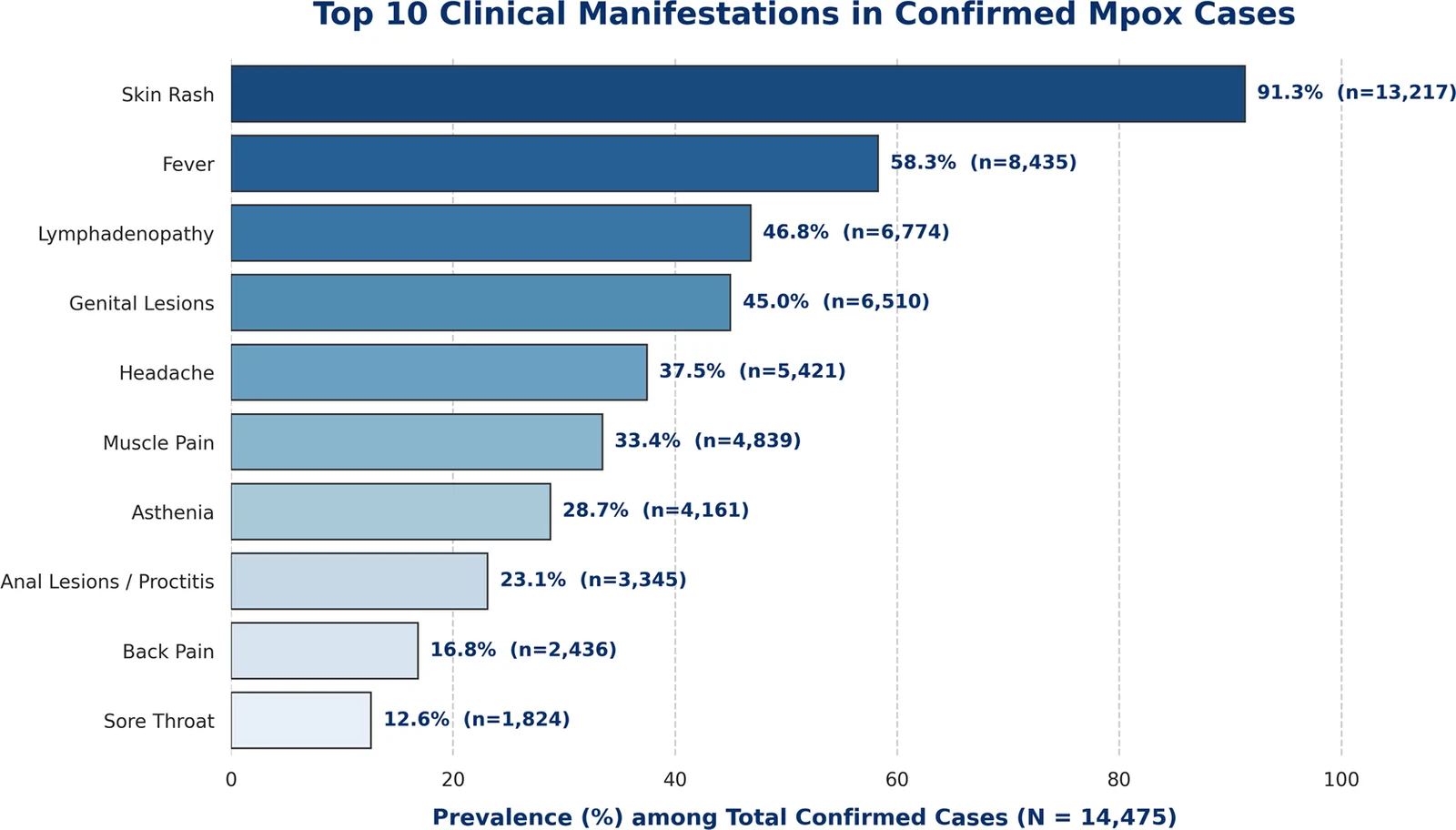
Prevalence of the most frequent clinical manifestations among laboratory-confirmed mpox cases in Brazil, 2022–2025 (*n* = 14 475). Data were obtained from OpenDataSUS.

Additional systemic symptoms included headache (37.5%; *n* = 5421), myalgia (33.4%; *n* = 4839) and asthenia (28.7%; *n* = 4161). Anorectal involvement (including anal lesions or proctitis) was reported in 23.1% of cases (*n* = 3345). Back pain (16.8%; *n* = 2436) and sore throat (12.6%; *n* = 1824) were less frequently observed (figure 3).

The clinical profile is characterized by a combination of mucocutaneous and systemic manifestations, with a notable frequency of genital and anorectal involvement.

## 4. Discussion

The 2022 global mpox outbreak represented a major shift in the epidemiology of the disease, which had previously been considered a rare zoonosis largely restricted to Central and West Africa [19]. The rapid international dissemination of MPXV, particularly the clade IIb lineage, resulted in sustained transmission across multiple non-endemic regions [5,13]. Brazil reported one of the highest case burdens globally, second only to the USA, reflecting its high population density and international connectivity. In Brazil, the initial epidemic wave in 2022 was characterized by a sharp increase in case numbers, followed by continued transmission in subsequent years, suggesting a transition from outbreak dynamics to more sustained patterns of circulation [14].

The spatial distribution of mpox cases in Brazil revealed a concentration of cases in the southeast and midwest regions, with São Paulo, Rio de Janeiro and Minas Gerais accounting for the largest number of reported cases. However, population-adjusted incidence rates demonstrated that transmission was not limited to the most populous states. States such as the Federal District and Amazonas exhibited elevated incidence relative to their population size, indicating heterogeneous transmission dynamics across regions. This pattern highlights the importance of integrating both absolute case counts and population-based metrics to identify areas of high transmission intensity and guide public health responses.

The demographic and behavioural profile observed in this study is consistent with global findings indicating a concentration of mpox cases among men, particularly those within groups of MSM. In our dataset, a high proportion of cases for which information on sexual orientation was available occurred among individuals who identify as homosexual, consistent with previous studies [15]. The substantial burden of HIV co-infection (approx. 47%) further reinforces the overlap between mpox transmission and existing networks of STIs. These findings highlight the need for targeted, non-stigmatizing public health interventions, focusing on prevention, testing and access to care within the affected populations [16–21].

Emerging evidence suggests that mpox infection may be associated with neuropsychiatric manifestations, including anxiety and psychological distress, which may result from both the clinical course of infection and the social context of isolation and stigma [22,23]. Previous studies have shown that demographic factors such as sex, education level and place of residence can influence awareness and risk perception related to mpox [24]. In Brazil, however, data on psychosocial outcomes remain limited. Although our dataset included basic demographic variables such as age and sex, it did not allow for a detailed assessment of mental health outcomes, highlighting an important gap for future research.

The clinical presentation observed in this study is consistent with previously reported mpox case series. Skin rash was the predominant manifestation, followed by fever and lymphadenopathy, reflecting the typical mucocutaneous and systemic profile of the disease. These findings are aligned with those reported by Thornhill *et al.* [22], who described cutaneous lesions in the majority of patients [25]. Although atypical presentations without rash have been documented, the high frequency of mucocutaneous involvement in our dataset reinforces its central role in clinical recognition and diagnosis.

Brazil's ecological diversity, demographic density and high degree of connectivity create conditions that facilitate the emergence and spread of infectious diseases [26]. These structural factors have previously contributed to the rapid dissemination of arboviruses such as Zika virus infection and the sustained transmission of dengue [27–29]. In this context, the persistence of mpox transmission highlights the importance of strengthening integrated surveillance systems and maintaining preparedness for emerging and re-emerging pathogens. Public health strategies should prioritize early detection, targeted interventions and equitable access to prevention and care to reduce transmission and mitigate broader dissemination.

This study has several limitations. First, it was based on secondary surveillance data from a national reporting system, which may be affected by underreporting, delayed notification, missing information and variability in data completeness across states and time periods. Second, several key variables, including sexual orientation and HIV status, had a substantial proportion of missing data, which may have influenced the observed distributions. Third, because the dataset was restricted to notified laboratory-confirmed cases, the findings likely underestimate the true burden of mpox, particularly among persons with limited access to healthcare or diagnostic services. Finally, the database did not include detailed information on behavioural exposures, clinical severity, treatment, viral lineage or long-term outcomes, limiting more refined analyses of transmission dynamics and prognosis.

## 5. Conclusion

This nationwide analysis of mpox in Brazil from 2022 to 2025 demonstrates a transition from an initial outbreak to sustained transmission. The epidemiological profile was characterized by a strong concentration of cases among adult males, particularly within sexual networks of MSM, and a high prevalence of HIV co-infection. These findings highlight the importance of integrated public health strategies that combine surveillance, testing and access to care, particularly within affected populations. Efforts should prioritize timely diagnosis, targeted prevention and non-stigmatizing communication to support effective outbreak control. The persistence of mpox transmission in Brazil underscores the need for sustained epidemiological monitoring and preparedness. Strengthening surveillance systems and ensuring equitable access to preventive measures, including vaccination where appropriate, will be essential to mitigate future transmission and limit broader dissemination.

## Supplementary Material



## Data Availability

This article has no additional data. Supplementary material is available online [30].
