# Supplementary material for: Epidemiological trajectory of Mpox in Brazil, 2022–2025: evidence of geographic consolidation and syndemic burden
Source: R Soc Open Sci. 2026 Oct 28;13(10):261003. doi: 10.1098/rsos.261003 (PMC13714395; doi:10.1098/rsos.261003)
Supplement: Supplementary file 1 [file rsos.261003.zip › rsos.261003/rsos261003_si_001.docx]

**Supplementary Table 1:** Incidence_final

**Supplementary_Figure1:** Figure
